# The effects of cognitive bias and cognitive style on trait impulsivity in moderate-risk gambling: The moderating effect of self-control

**DOI:** 10.3389/fpsyg.2023.1089608

**Published:** 2023-01-27

**Authors:** Wenwen Shi, Na Li

**Affiliations:** School of Physical Education, Hubei University, Wuhan, China

**Keywords:** cognitive bias, cognitive style, trait impulsivity, self-control, moderate risk gambling

## Abstract

**Background:**

Impulsivity has been defined as a tendency to respond with little forethought, often with disregard to the negative consequences to the impulsive individual or others. Problem gambling patients are characterized with impulse control and absent inhibition control, a tendency to react to stimuli in a rapid and unplanned fashion without complete processing of information.

**Method:**

Based on the information processing theory and the dual-systems model of self-control, 208 moderate-risk gambling were investigated by questionnaire to explore the moderating effect of self-control in the process of cognitive bias and cognitive style affecting the gambling impulse of moderate-risk gambling.

**Conclusion:**

Using hierarchical regression analysis, it is found that: (a) The gambling impulse of male moderate-risk gambling was stronger than female moderate-risk gambling. (b) Self-control negatively predicted trait impulsivity, and the stronger the individual self-control, the lower the level of trait impulsivity. (c) Cognitive bias positively predicted trait impulsivity, and high cognitive bias induced high-level trait impulsivity. Self-control played a moderating role between cognitive bias and trait impulsivity. (d) Compared with field-independent gambling, field-dependent gambling were more likely to have impulsive thoughts of gambling activities. Self-control played a moderating role between cognitive style and trait impulsivity.

## Introduction

Problem gambling, understood as experiencing negative consequences of using gambling services, is prevalent in 0.12–5.8% of the population in all the parts of the world, and is recognized as a public health issue in many countries (Ivanova et al., 2019). Problem gambling has been defined as a biopsychosocial disorder characterized by a persistent and recurrent maladaptive pattern of gambling behavior (Ssewanyana and Bitanihirwe, 2018). Included in the spectrum of addictive disorders in the 5th version of the Diagnostic and Statistical Manual of Mental Disorders (DSM-5), Problem gambling shares many similarities with substance use disorders, at the behavioral, psychological, and neurobiological level (Challet-Bouju et al., 2017).

The Canadian Centre for Substance Abuse Research views gambling as a gradual behavior that occurs along a continuum, not a simple two-dimensional (normal behavior and problem behavior). The harm caused by individual gambling behavior to themselves, others, and society determines the position of gambling along this continuum. To this end, the Center specially developed a measurement questionnaire, namely the Problem Gambling Severity Index (PGSI), which refines the categories of individuals in the whole continuum. The PGSI classified gambling into non-problem gambler, low-risk gambler, moderate-risk gambler, and problem gambler (Boldero and Bell, 2011). Very few individuals immediately fit into a ‘problem gambling’ category, but rather gradually progress from non-problem to problematic or risky gambling behaviors (Thomas et al., 2013).

Moderate-risk gambling can develop in three trends: first, suspend gambling activities; second, continue to gambling activities, but do not fall into it, and change themselves from moderate-risk gambling to low-risk or non-problem gambling; third, continue to gambling activities, but deeply trapped in it, and change themselves from moderate-risk gambling to problem gambling. Although the moderate-risk gambling did not reach the problem gambling through the scale diagnosis, they were very likely to suffer the negative consequences of gambling or facing the risk of evolving into problem gambling. While in the current research, more attention has been paid to the ordinary gambling (Welte et al., 2002) and the problem gambling (Donati et al., 2018; Shi et al., 2021). At present, in the study of problem gambling, more attention is paid to the structural characteristics of the research group (Cowie et al., 2017; Hu et al., 2017) and intervention measures (Leung and Cottler, 2009), while less attention is paid to the prevention research on problem gambling, especially how to effectively identify the key populations with high susceptibility and addiction tendency. Lyk-Jensen (2010) argued that preventing individuals from evolving into problem gambling depends on the in-depth analysis of moderate-risk gambling, and the prevention object should take moderate-risk gambling as the breakthrough. Therefore, what are the core factors that affect the transformation from moderate-risk gambling to problem gambling?

Impulsivity has been defined as a tendency to respond with little forethought, often with disregard to the negative consequences to the impulsive individual or others (Mestre-Bach et al., 2020). According to this definition, researchers define trait impulsivity as the psychological tendency of sports gambling to make rapid, unconsidered, and unplanned responses to internal or external stimuli in the process of sports gambling consumption, regardless of whether these reactions will have negative consequences for individuals, families, organizations, and society.

In the research of behavioral addiction, scholars pointed out that individual trait impulses will lead to the occurrence of problem psychology and behavior. Impulsivity is an unplanned action tendency, which plays an essential role in the development and maintenance of addictive behavior (Verdejo-García et al., 2008). Impulsivity is likewise considered as a central issue in the consumption of sports lottery. The American Psychiatric Association (APA) defines pathological gambling as an impulsive control disorder, and impulsivity is considered to be the most significant risk factor on influencing factors for gambling addiction among many studies. Shenassa et al. (2012) conducted a follow-up survey of 958 children for 30 years, and found that impulsive behavior which exhibited at the age of 7 would become a risk factor for his gambling in adulthood. Stautz and Cooper (2013) also found that impulsivity level in adolescence can predict substance addiction behavior in adulthood. Goudriaan et al. (2005) used GO/NOGO task to investigate individual behavior inhibition ability, and found that gambling addicts could not effectively control themselves, resulting in higher error rate and lower response inhibition ability, indicating a high impulsivity. The above findings not only reflect the positive correlation between impulsivity and addictive behavior, but also provide an idea for this study that impulsivity is potentially a core element in the transition of risk gambling. Lai et al. (2011) synthesized previous research results and believed that the trait impulse of gambling was regarded as a personality trait with cross-situational consistency and cross-time stability. The previous research results also found that the impulsivity of gambling plays a crucial role in the change of gambling behavior. Hence, what elements have an important influence on gambling purchase impulsivity? What is the relationship between these influencing factors? This study attempted to answer the above questions from the perspective of information processing theory and the dual-systems model of self-control.

## Theoretical basis

### Information processing theory

Individual behavioral decision-making relied on the understanding of stimulus information, that is, individual cognition played an important role in the consumption progress. Human cognition began with external stimuli, went through the steps of information perception and information processing, and finally reached the reaction stage. Among them, the information perception and information processing together constituted the individual perception process. Information perception is the process of human sensory organs receiving and perceiving external information (distal stimuli). In contrast, information processing identifies the perceived information (proximal information), gives it a particular interpretation and meaning, and classifies it as a kind of object or event. The reaction stage is the stage of reacting to processed information with two main functions: (a) Thinking about how to deal with the identified stimuli (information), making the best decision, and putting it into practice. (b) Information is transformed into knowledge structure and stored to form working (short-term) memory and long-term memory.

Based on the theory of information processing, we can analyze the decision-making process of gambling as follows: the decision-making response of gambling may originate from information dissemination of gambling advertising, peer persuasion, and people’s material values. The individual perception system accepts external advertising information (distal information) and converts it into perceived advertising information (proximal information). Peer persuasion is an important source of information, and social learning theory holds that people readily learn and recognize the attitudes of important others toward external events. Peer group’s perceptions that “gambling is profitable” and that “gambling can lead to rich,” and so on, influence others. This link belongs to the first stage of perception-information perception. Then, when gambling enter the second stage of perception-information processing, they will input perceptual information for processing. Cognitive style is an individual difference in information processing. Individual information processing ability will be affected by personality and cognitive characteristics, therefore, there will be different psychological and behavioral reactions between gambling in the final reaction stage.

In conclusion, the process of gambling purchase is essentially an information processing process. The information processing theory presents a road map from external stimulation information, perception information of gambling, processing and editing information, thinking and decision-making to impulsive response and emotional response, and finally forming gambling behavior.

### The dual-systems model of self-control

The Dual-systems model of self-control was jointly proposed by Hofmann et al. (2009) in 2009, which provided a new perspective for interpreting individual behavioral biases. They argued that self-control was essentially a process of resisting impulsivity. When people were faced with external temptation, there were two forces: one was the impulsive force induced by individual internal desire and the other was the self-control force that warned individuals to make rational decisions. These two forces are the impulse system and the self-control system. The impulsive system is an important reason for individuals to make hasty decisions, and the occurrence of impulsivity is an automatic response of individuals to temptation stimuli, including automatic emotional reactions and automatic approach-avoidance responses (Seibt et al., 2007). Impulse processing is an automated form formed over time without individual input of attention resources (Gawronski and Bodenhausen, 2006). The self-control system is the cause of higher-order psychological activities generated when individuals are exposed to temptation and stimuli, including deliberate appraisal and inhibition criteria (Strack and Deutsch, 2004). Friese et al. (2008) demonstrated that the above two kinds of high-order psychological activities depended on self-control processing. The theory also pointed out that the contestation and coordination between impulse power and self-control power could be regulated by other variables, including state and trait regulation variables. The state regulation variables were mainly self-control resources and alcohol consumption. The main trait moderating variables were working memory capacity and trait self-control.

**Figure 1:**
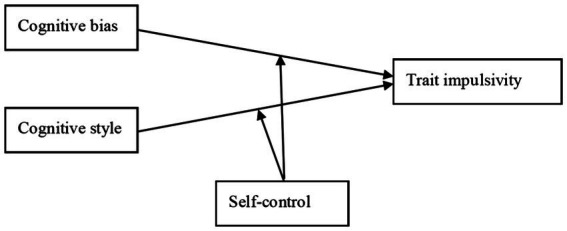
Theoretical method.

In summary, individual cognitive characteristics and self-control will have an important impact on trait impulsivity. However, we are not clear about the interaction among cognitive bias, cognitive style, self-control, and trait impulsivity. Therefore, the present study intends to investigate the mechanisms of cognitive bias, cognitive style, and self-control on trait impulsivity.

### Hypotheses

Cognitive bias is the distorted perception of objective facts produced by individuals in the process of social cognition. Cognitive psychology believes that the human brain’s perception of external information is limited, people tend to ignore information too fast or too much, and perceive more specific or vital information. Meanwhile, the selective memory of human beings leads to only the high frequency information, and outstanding features can be easily remembered. Therefore, In the process of gambling, winners may experience the perceptual luck of “change of fortune,” while losers may experience the sunk cost effect of “seize the opportunity.” Scholars have also confirmed the objective fact that gambling have cognitive bias. The cognitive theory of gambling emphasizes the gambling’ irrational beliefs at the different stages of their activities. The main irrational beliefs are gambling’ fallacy, entrapment, belief in hot and cold numbers, unrealistic optimism or perceived luckiness, superstitious belief, illusion of control, near miss, and roll over effect. The following paragraphs will briefly review these beliefs and provide some illustrations (Ariyabuddhiphongs, 2011). Mackillop et al. (2006) measured the problem gambling behavior among college students and found a positive association between Eysenck Impulsivity Scale scores and Gambling Belief Scale scores. Mobini et al. (2007) also reported the relationship between impulsivity and cognitive bias and reached consistent conclusions. Michalczuk et al. (2011) used the UPPS-P and GRCS to measure trait impulsivity and cognitive bias. The results showed that the phenomenon of cognitive bias of problem gambling was relatively serious, and there was a high level of impulsivity, especially in the dimensions of positive urgency and negative urgency.

Cognitive style refers to the habitual way in which individuals show preference when organizing and representing information, and it is the individual difference in people’s perception, thinking, learning, and problem solving (Witkin, 1965). Field-dependent individuals tend to determine their own attitudes, feelings, and behaviors based on external information as a frame of reference. Field-dependent individuals are more sensitive to social situational factors and more vulnerable to situational factors. Field-independent individuals tend to take the internal as the reference frame, do not actively process the environmental clues, and are less affected by situational factors.

Literature review showed that the research on cognitive style and impulsivity was minimal. However, we can learn from scholars’ exploration of the relationship between cognitive style and personality traits. Deng et al. (2000) found that field dependence was significantly negatively correlated with emotional stability and boldness. The stronger the field dependence, the worse the emotional stability of the individual, the more impulsive the individual, and the more adventurous the individual. Cognitive style has a certain influence on individual impulsivity, and field-dependent gambling are more likely to stimulate individual idiosyncratic impulsivity. The occurrence of behavioral impulsivity is caused by the failure of individual to resist external temptation, that is, the failure of self-control leads to impulsivity. Some scholars believe that the self-control is the ability to regulate, manipulate and control personal impulsive thoughts, emotions and behaviors. Duckworth and Kern (2011) noted that self-controlled individuals could better regulate their attention, emotion, and behavioral impulsivity than impulsive individuals. In conclusion, self-control has two characteristics: it can actively regulate individual cognition, emotion, and behavior; moreover, it is also a psychological function of resisting temptation and restraining impulse to achieve long-term goals. In short, self-control plays an important moderating role in individual social life. In the process of gambling, gambling think that when they watch a certain football match (illusion of control), their internal impulse will drive them to purchase a large amount of lottery, but lack of money and persuasion from others may inhibit their gambling behavior, and then the internal conflict will occur. Dual-systems model of self-control points out that when two forces are in conflict, they are regulated by self-control.

In summary, this study will investigate the effects of cognitive bias, cognitive style, and self-control on trait impulsivity based on information processing theory and dual-systems model of self-control. Moreover, this paper intends to take self-control as a third-party variable to investigate the mechanism of cognitive bias and cognitive style on trait impulsivity. Accordingly, this paper constructed the theoretical model as shown in Figure 1.

## Materials and methods

### Subjects screening

According to the distribution of administrative regions, select one province in the East, West, South, North, and central regions, respectively. The specific provinces are Fujian Province, Hubei Province, Jiangsu Province, Chongqing city, and Liaoning Province. Two thousand two and thirty questionnaires were distributed to the sports lottery sales stores in the above provinces, respectively. Two thousand one hundred and twelve questionnaires were collected, and a total of 2025 valid questionnaires were retained. Among them, there were 1,698 males and 327 females, aged 19–71 years. The subjects were tested with the Problem Gambling Severity Index (PGSI) to screen 1,451 non-problem gambling, 323 low-risk gambling, 213 moderate-risk gambling, and 29 problem gambling. Beck Depression Inventory was used to exclude subjects with moderate depression with a score of 23. Anxiety Self-Rating Inventory was used to exclude subjects with moderate anxiety (60–69) and severe anxiety (70–79). Finally, 208 moderate-risk gambling were obtained.

### Research tool

#### Self-control scale

The self-control scale measures dispositional self-regulatory behaviors using 12 items rated on a 5-point scale, ranging from 1 (Not at all like me) to 5 (Very much like me). The reliability and validity test of this survey showed that the Cronbach α of the scale was 0.859. The results of structural validity were: *χ*^2^ = 172.15, df = 54, *χ*^2^/df = 3.18, SRMR = 0.077, NFI = 0.81, NNFI = 0.81, CFI = 0.84, IFI = 0.84.

#### Barratt impulsiveness scale

The Barratt Impulsiveness Scale (BIS-11) is designed to measure the personality trait of impulsivity in this study. The scale consists of 30 items, and the participant is requested to answer each on a 4-point Likert scale from 1(rarely/never) to 4(almost always/always). These fell under three second-order factors: attentional impulsiveness, motor impulsiveness, and non-planning impulsiveness. The reliability and validity test of this survey showed that the Cronbach α of the scale was 0.861, including the non-planning impulsiveness subscale of 0.878, the motor impulsiveness subscale of 0.892, and attentional impulsivity subscale of 0.883. The results of structural validity of the questionnaire are as follows: *χ*^2^ = 1005.52, df = 402, *χ*^2^/df = 2.50, SRMR = 0.079, NFI = 0.90, NNFI = 0.90, CFI = 0.92, IFI = 0.92.

#### Cognitive style test

In this study, the Embedded Figure Test (EFT), revised by the Psychology Department of Beijing Normal University, was used to measure cognitive style (Including field-independent and field-dependent). The scale consists of four parts, the first part is 9 simple figures, the second part is 9 practice figures, and the third and fourth parts are a total of 20 formal test figures. The subjects need to overcome the interference effect of the negative complex figure and find the simple figure within the complex figure. The figure found must be completely consistent with the specified simple figure in size and direction to be correct. A correct answer is worth 1 point, with a maximum score of 20 points. The higher the score, the stronger the field-independent of the subjects; the lower the score, the stronger the field-dependent of the subjects. The reliability and validity test of this survey showed that the Cronbach α of the scale was 0.821.

#### Gambling purchase cognitive bias questionnaire

Gambling purchase cognitive bias questionnaire consists of 8 items rated on a 5-point scale, ranging from 1 (Not at all like me) to 5 (Very much like me). The confirmatory factor analysis of this survey showed that *χ*^2^ = 24.40, df = 17, RMSEA = 0.052, SRMR = 0.036, NFI, GFI, CFI, and IFI were all above 0.90, indicating that the three-factor structure model fitted well and could be used as an investigation tool. Internal consistency reliability (*α* coefficient) was used as a reliability indicator in this study. It is found that the internal consistency (*α* coefficient) of the dimensions was good: almost winning (*α* = 0.857), the illusion of control (*α* = 0.890), and sunk cost (*α* = 0.866), indicating a high reliability of the questionnaire.

## Results

### Differences of demographic variables in trait impulsivity

In this study, trait impulsivity and three sub-dimensions were used as dependent variables, and the gender of moderate-risk gambling was used as independent variables for variance analysis. The test results are as follows: the trait impulse level of male moderate-risk gambling (*M* = 71.987, SD = 7.011) was higher than that of female moderate-risk gambling (*M* = 68.77, SD = 8.637), and there was a significant difference (*F* = 7.022, *p* < 0.01). The results also showed that (see Table 1) there were significant gender differences in the two sub-dimensions of non-planning impulsiveness (*F* = 5.371, *p* < 0.05) and motor impulsiveness (*F* = 6.873, *p* < 0.01), and there was no significant difference in attentional impulsiveness (*F* = 0.211, *p* > 0.05; see Table 1).

**Table 1:** Gender difference test of trait impulsivity.

Variable	Male (*N* = 160)	Female (*N* = 48)	*F*	*p*
	(M ± SD)	(M ± SD)		
Non-planning impulsiveness	27.57 ± 4.151	25.92 ± 4.894	5.371*	0.021
Motor impulsiveness	23.81 ± 2.578	22.58 ± 3.619	6.873**	0.009
Attentional impulsiveness	20.61 ± 4.087	20.27 ± 5.441	0.211	0.646
BIS-11 total score	71.987 ± 7.011	68.77 ± 8.637	7.022**	0.009

Numbers represent One-Way ANOVA. ^*^ indicates *p* < 0.05, ^**^ indicates *p* < 0.01.

In this study, trait impulsivity and three sub-dimensions were used as dependent variables, and the age of moderate-risk gambling was used as independent variables to conduct variance analysis. The test results are as follows: there were no significant differences in the total score of BIS-11, non-planning impulsiveness, motor impulsiveness, and attentional impulsiveness among moderate-risk gambling of different ages (see Table 2).

**Table 2:** Age difference test of trait impulsivity.

Variable	Under 18 years old (*N* = 37)	19–29 years old (*N* = 88)	30–39 years old (*N* = 56)	Over 40 years old (*N* = 27)	*F*	*p*
	(M ± SD)	(M ± SD)	(M ± SD)	(M ± SD)		
Non-planning impulsiveness	26.62 ± 4.615	27.34 ± 4.533	27.05 ± 4.346	27.74 ± 3.665	0.400	0.753
Motor impulsiveness	23.46 ± 3.355	23.52 ± 2.771	24.09 ± 2.560	22.48 ± 3.081	1.918	0.128
Attentional impulsiveness	20.22 ± 5.308	21.08 ± 4.286	20.11 ± 3.784	20.04 ± 4.824	0.794	0.498
BIS-11 total score	70.30 ± 6.679	71.94 ± 8.245	71.25 ± 6.810	70.26 ± 7.383	0.606	0.612

The trait impulsivity and three sub-dimensions were used as dependent variables, and the educational background of moderate-risk gambling was used as independent variables to conduct variance analysis. The test results are as follows: there was no significant difference in the total score of BIS-11, non-planning impulsiveness, motor impulsiveness, and attentional impulsiveness among moderate-risk gambling with different educational backgrounds (see Table 3).

**Table 3:** Educational background difference test of trait impulsivity.

Variable	Below high school (*N* = 90)	College and undergraduate (*N* = 82)	Postgraduate (*N* = 36)	*F*	*p*
	(M ± SD)	(M ± SD)	(M ± SD)		
Non-planning impulsiveness	27.00 ± 4.370	27.40 ± 4.494	27.17 ± 4.219	0.180	0.835
Motor impulsiveness	23.46 ± 3.134	23.66 ± 2.405	23.42 ± 3.307	0.138	0.872
Attentional impulsiveness	20.62 ± 4.796	20.85 ± 4.116	19.56 ± 4.102	1.113	0.330
BIS-11 total score	71.08 ± 7.748	71.91 ± 7.113	70.14 ± 7.684	0.742	0.477

### Correlation analysis of variables

See Table 4 for the mean, standard deviation, and correlation coefficients between the dimensions of cognitive bias, cognitive style, self-control, and trait impulsivity. It can be seen that there was a significant positive correlation between cognitive bias and trait impulsivity (*r* = 0.600), a significant negative correlation between cognitive style and trait impulsivity (*r* = −0.285), a significant negative correlation between self-control and trait impulsivity (*r* = −0.573), and a significant negative correlation between self-control and cognitive bias (*r* = −0.247). There was a significant negative correlation between self-control and cognitive style (*r* = −0.233).

**Table 4:** Mean value, standard deviation of each variable, and correlation coefficient between each dimension (*N* = 208).

	Mean	SD	1	2	3	4
Self-control	34.52	6.714	1			
Cognitive bias	26.92	6.978	−0.277^**^	1		
Cognitive style	15.06	3.413	0.237^**^	−0.182^**^	1	
Trait impulsivity	71.25	7.483	−0.629^**^	0.584^**^	−0.285^**^	1

Numbers represent pearson’s correlations. ^**^ indicates *p* < 0.01.

### Regression analysis

In order to exclude the influence of demographic variables such as gender, age, and educational background, three demographic variables were included in the statistical analysis as control variables in the test of the moderating effect of self-control. Subsequently, hierarchical regression analysis was used to investigate the moderating role of self-control in the effects on cognitive bias and cognitive style on trait impulsivity. There are three steps in the analysis. First, cognitive bias (independent variable), cognitive style (independent variable), and self-control (moderating variable) are first centralized to avoid covariance problems. Second, cognitive bias, cognitive style, and self-control were multiplied as interactive items. Third, after controlling for demographic variables, cognitive bias, cognitive style, self-control, and their interaction items were successively included in the regression equation. The research results are shown in Table 5.

**Table 5:** Hierarchical regression analysis results of cognitive bias, cognitive style, self-control, and trait impulsivity.

Mode	Predictive variable	Step 1	Step 2	Step 3
Model 1	Gender	−0.197^**^	−0.132^*^	−0.115^*^
	Age	−0.1	−0.066	−0.052
	Education	−0.014	0.013	0.047
Model 2	Cognitive bias		0.536^***^	2.021
	Cognitive style		−0.180^**^	1.374
Model 3	Self-control			1.507
	Cognitive bias × Self-control			−2.522^*^
	Cognitive style × Self-control			−3.023^*^
	Cognitive bias × Cognitive style			−1.741
	Cognitive bias × Cognitive style × Self-control			2.919
	*R* ^2^	0.044	0.392	0.624
	Adjusted *R*^2^	0.03	0.377	0.605
	DR^2^	0.044	0.348	0.232
	*F*	3.103^*^	26.096^**^	32.739^**^

Numbers represent hierarchical regression analysis. ^*^ indicates *p* < 0.05, ^**^ indicates *p* < 0.01.

It can be seen from Tables 4, 5 that cognitive bias positively predicted the trait impulse of moderate-risk gambling (*β* = 0.536, *p* < 0.001), cognitive style negatively predicted the trait impulse of moderate-risk gambling (*β* = −0.180, *p* < 0.01), the interaction of cognitive bias and self-control had a significant negative prediction on the trait impulse of gambling (*β* = −2.522, *p* < 0.05), and the interaction of cognitive style and self-control had a significant negative prediction on the trait impulse of gambling (*β* = −3.023, *p* < 0.05).

However, the interaction of cognitive bias and cognitive style, as well as the triple interaction term of cognitive bias, cognitive style, and self-control, did not significantly predict the trait impulsivity of gambling, indicating that self-control played a significant role in the regulation of cognitive bias and trait impulsivity. To further analyze the specific role of moderating variables, a low self-control group (low subgroup) and a high self-control group (high subgroup) were obtained according to the specified proportion in statistics of 27% and then, a simple slope test analysis was conducted.

**Figure 2:**
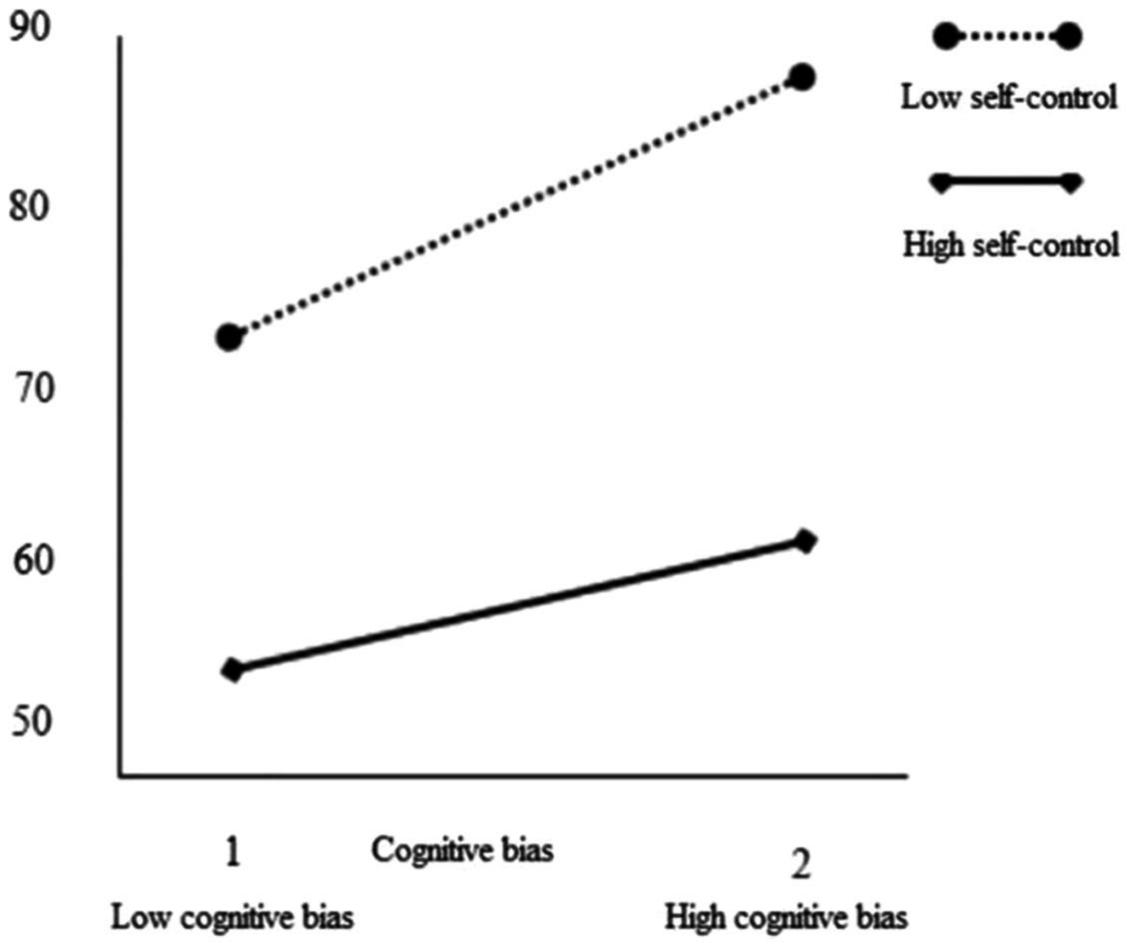
The moderating role of self-control between cognitive bias and trait impulsivity.

**Figure 3:**
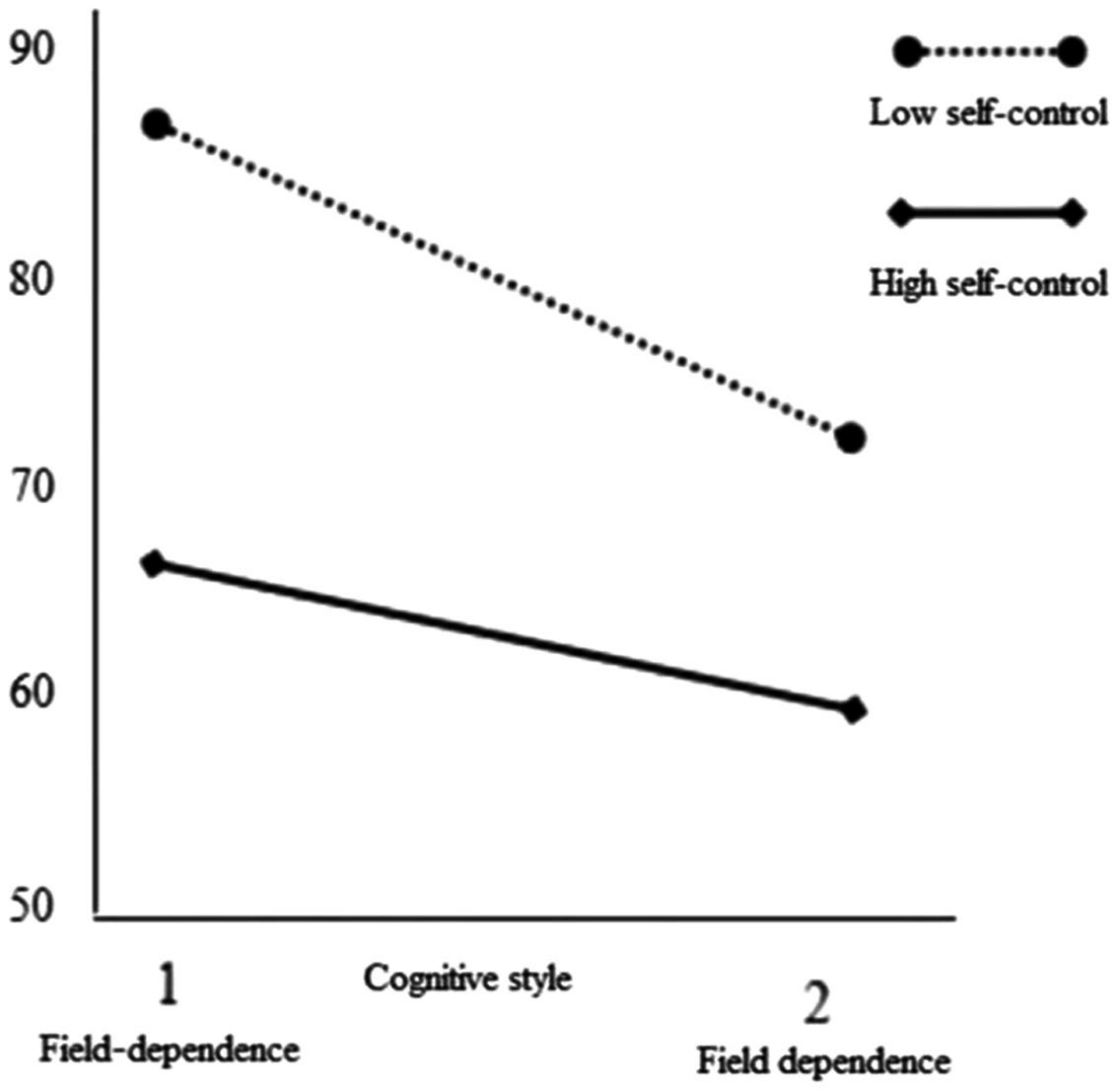
The moderating role of self-control between cognitive style and trait impulsivity.

Meanwhile, the interaction diagrams of self-control and cognitive bias and self-control and cognitive style were drawn, respectively, as plotted in Figures 2, 3. Self-control had the moderating effect on cognitive bias and trait impulsivity. Under a certain level of cognitive bias, self-control had an obvious moderating effect. When the moderate-risk gambling were in low self-control, their trait impulse would increase significantly with the increase of the level of cognitive bias. When the moderate-risk gambling were in high self-control, their trait impulsivity also changed with the increase of cognitive bias, but its growth rate was far lower than that in low self-control.

Self-control had the moderating effect on the relationship between cognitive style and trait impulsivity. When the moderate-risk gambling were in low self-control, the trait impulse level of field-dependent gambling was much higher than that of field-independent gambling. When the moderate-risk gambling were in high self-control, there was no significant change in the level of trait impulse between field-independent gambling and field-dependent gambling.

## Discussion

### Gender differences in trait impulsivity

The results showed that the scores of male moderate-risk gambling were higher than those of female moderate-risk gambling. There were significant gender differences in the two sub-dimensions of non-planning impulsiveness (*F* = 5.371, *p* < 0.05) and motor impulsiveness (*F* = 6.873, *p* < 0.01), but there was no significant difference in attentional impulsiveness (*F* = 0.211, *p* > 0.05). In other words, compared with female gambling, male gambling may lack of planning in the gambling process, and are prone to trait impulsivity on the spur of the moment and they are reckless. The theory of gender socialization holds that males and females are endowed with different value orientations, role expectations, behavior patterns, and personality traits in the process of socialization. The society gives males and females different reinforcement and encouragement based on specific role needs. Females are more inclined to compliance and care, and males are inclined to achieve goals and external returns. Therefore, male risk gambling are more likely to adopt impulsive gambling behavior in pursuit of material rewards. In addition, from the perspective of evolutionary psychology, impulsive gender differences, males have stronger risk acceptance than females, and have a stronger risk-taking tendency (Wilson and Daly, 1985).

### Cognitive bias and trait impulsivity: The moderating role of self-control

The results showed that cognitive bias positively predicted trait impulsivity, and high cognitive bias induced high-level trait impulsivity. Further stratified regression analysis showed that self-control played a moderating role between cognitive bias and trait impulsivity.

Researchers have suggested that the illusion of control (i.e., where gambling falsely over-estimate their ability to influence outcomes), is higher for individuals who participate in skill-based gambling (Cantinotti et al., 2004). It is the limitations of human beings themselves that individuals are prone to bias in cognitive decision-making, especially in sports bettors. Li (2011) explored the influencing factors of unhealthy psychology in gambling based on the theory of communication. The results showed that a large number of professional, systematic, and accurate dissemination of gambling information could strengthen the cognitive psychology of the gambling to get something for nothing and control the illusion. In the process of gambling, when the realistic results are consistent with the perceived information, gambling may have illusion of control (combining expert information and a “unique secret” to win the gambling). When the real result is inconsistent with the perceived information, cognitive deviation phenomena may occur, as an illustration, there may be a near-win (just one game away from winning jackpot/wrong number, the first prize is reduced to the third prize) and sunk costs (continue to bet to recover the principal/this group of numbers continue to bet, otherwise all the money has been wasted). Whether consistent or not, the cognitive bias of gambling seems to have some kind of “positive” results (winning the grand prize 1 day), which often stimulates their positive emotions such as excitement, expectation, and hope. Such positive emotions make gambling overly optimistic about their own choices and winning probability, resulting in emotional impulse gambling (Wright and Bower, 1992).

It has been found that individuals with high self-control tended to seek information and situations that were likely to trigger impulsivity more than individuals with low self-control (Tangney et al., 2004). In this study, we found that self-control negatively predicted trait impulsivity and moderated the relationship between cognitive bias and trait impulsivity. Moreover, cognitive bias had a greater effect on low self-control participants. Self-control played an important moderating role in individual social life. When the self-control ability was low, with the enhancement of cognitive bias, the impulsive gambling behavior became more prominent. When the self-control ability was high, with the enhancement of the level of cognitive bias, the impulsive gambling behavior would increase correspondingly, but it was generally lower than that of gambling with low self-control ability. This showed that in the process of sports bettors, the self-control ability of gambling did have an important impact on their impulsive gambling behavior. Compared with the low self-control gambling, the high self-control gambling could better resist the external “positive” information, even if such information caused by the illusion of control, almost win and other cognitive biases, they might quickly make adjustments to their thinking and suppress their trait impulsivity. On the contrary, low self-control gambling might be weak in ability to resist temptation, easy to be induced by bad information. If gambling appear more serious cognitive bias, the two forces will greatly promote their trait impulsivity.

### Cognitive style and trait impulsivity: The moderating role of self-control

The study found that compared with field-independent subjects, field-dependent subjects were more likely to have impulsive thoughts of gambling. Self-control negatively predicted trait impulsivity. The stronger the individual self-control, the lower the level of trait impulsivity. Further hierarchical regression analysis showed that self-control played a moderating role between cognitive style and the trait impulse of gambling.

The results of the cognitive style test showed that the field-independent subjects can find simple graphics from complex graphics, indicating that they have stronger cognitive reorganization ability. In the process of sports gambling consumption, compared with field-dependent gambling, field-independent gambling can extract from the numerous advertising information and build the internal relationship of information, so as to grasp and understand the essence of things. In addition, the field-independent gambling have the mature meta-cognitive ability. They conduct self-awareness, self-reflection, and self-adjustment in their own cognitive processing. When they are aware of the “invasion” of bad information, they will timely adjust their gambling strategies to restrain impulsive gambling. While the field-dependent gambling are different. They are usually more sensitive to external information, more dependent on information, but the working memory span of people is limited, and field-independent gambling in the information explosion environment will produce cognitive overload. At this time, they may only focus on the distinctive and attractive information, and some important vigilant information will be discarded, which destroys the construction of the internal relationship of gambling information, so it is easy to be affected by bad information, driven by money motivation, trait impulsivity appears. Individuals have different ways of processing information, and cognitive style is the consistent way of organizing and processing information (Tennant, 1988). Therefore, under the joint action of different cognitive styles and different levels of self-control, individuals will produce specific psychological reactions. According to the interaction diagram, for field-dependent gambling, the trait impulsivity level was higher under the influence of low self-control, while lower under the influence of high self-control, suggesting that the field-dependent gambling were not unable to resist the invasion of sudden wealth information. As long as they have a high level of self-control, it can effectively inhibit the impulse to purchase gambling. Similarly, the field-independent gambling cannot maintain rational gambling, but also produce a higher level of trait impulsivity under the action of low self-control. Self-control has the ability to actively regulate individual cognition, emotion, and behavior, and the field-dependent gambling are more sensitive to external stimuli, therefore, lower self-control tends to stimulate more impulsive gambling behaviors.

## Conclusion

The gambling impulse of male moderate-risk gambling was stronger than female moderate-risk gambling. Self-control negatively predicted trait impulsivity, and the stronger the individual self-control, the lower the level of trait impulsivity. Cognitive bias positively predicted trait impulsivity, and high cognitive bias induced high-level trait impulsivity. Self-control played a moderating role between cognitive bias and trait impulsivity. Compared with field-independent gambling, field-dependent gambling were more likely to have impulsive thoughts of gambling purchase. Self-control played a moderating role between cognitive style and trait impulsivity. In conclusion, trait impulsivity with different cognitive characteristics (cognitive biases and cognitive styles) is regulated by self-control ability. How to enhance individual self-control ability will become the core content of future prevention programs.

## Data availability statement

The original contributions presented in the study are included in the article/supplementary material, further inquiries can be directed to the corresponding author.

## Ethics statement

The studies involving human participants were reviewed and approved by all observers who provided informed written consent. The study was approved by the institutional review board (ethics committee) of the School of Physical Education at Hubei University. The patients/participants provided their written informed consent to participate in this study.

## Author contributions

WS contributed to the conception, design of the study, and wrote the first draft of the manuscript. NL wrote sections of the manuscript. All authors contributed to manuscript revision and read and approved the submitted version.

## Funding

This research was supported by grants from the Humanities and Social Science Fund of Ministry of Education of China (21YJC890027) and the Educational Commission of Hubei Province of China (20D009) and the Academic Innovation Team Project for Young Scholars of Hubei University in 2022 (HBQN0107).

## Conflict of interest

The authors declare that the research was conducted in the absence of any commercial or financial relationships that could be construed as a potential conflict of interest.

## Publisher’s note

All claims expressed in this article are solely those of the authors and do not necessarily represent those of their affiliated organizations, or those of the publisher, the editors and the reviewers. Any product that may be evaluated in this article, or claim that may be made by its manufacturer, is not guaranteed or endorsed by the publisher.
